# Imaging of the Entry Pathway of a Cell-Penetrating Peptide–DNA Complex From the Extracellular Space to Chloroplast Nucleoids Across Multiple Membranes in *Arabidopsis* Leaves

**DOI:** 10.3389/fpls.2021.759871

**Published:** 2021-12-03

**Authors:** Kazusato Oikawa, Ayaka Tateishi, Masaki Odahara, Yutaka Kodama, Keiji Numata

**Affiliations:** ^1^Department of Material Chemistry, Graduate School of Engineering, Kyoto University, Kyoto, Japan; ^2^Biomacromolecules Research Team, RIKEN Center for Sustainable Resource Science, Wako, Japan; ^3^Center for Bioscience Research and Education, Utsunomiya University, Utsunomiya, Japan

**Keywords:** *Arabidopsis thaliana*, cell-penetrating peptide, DNA delivery, chloroplast nucleoid, field emission-scanning electron microscope, confocal laser scanning microscopy, plasma membrane, cell wall

## Abstract

Each plant cell has hundreds of copies of the chloroplast genome and chloroplast transgenes do not undergo silencing. Therefore, chloroplast transformation has many powerful potential agricultural and industrial applications. We previously succeeded in integrating exogenous genes into the chloroplast genome using peptide–DNA complexes composed of plasmid DNA and a fusion peptide consisting of a cell-penetrating peptide (CPP) and a chloroplast transit peptide (cpPD complex). However, how cpPD complexes are transported into the chloroplast from outside the cell remains unclear. Here, to characterize the route by which these cpPD complexes move into chloroplasts, we tracked their movement from the extracellular space to the chloroplast stroma using a fluorescent label and confocal laser scanning microscopy (CLSM). Upon infiltration of cpPD complexes into the extracellular space of *Arabidopsis thaliana* leaves, the complexes reached the chloroplast surface within 6h. The cpPD complexes reached were engulfed by the chloroplast outer envelope membrane and gradually integrated into the chloroplast. We detected several cpPD complexes localized around chloroplast nucleoids and observed the release of DNA from the cpPD. Our results thus define the route taken by the cpPD complexes for gene delivery from the extracellular space to the chloroplast stroma.

## Introduction

Chloroplasts have a small genome, the remnants of the endosymbiotic cyanobacterium genome. The chloroplast genome is 120–200kb depending on the plant species, and is found inside the chloroplast stroma in a compact form termed the nucleoid (Sakamoto, 2006). Leaf mesophyll cells from the land plant *Arabidopsis* (*Arabidopsis thaliana*) have about 100 chloroplasts, each containing ∼50 copies of the chloroplast genome; therefore, the chloroplast DNA in a cell is almost two orders of magnitude more abundant than the nuclear genome (Jarvis and Lopez-Juez, 2013). Transgenes incorporated into the chloroplast genome can therefore reach much higher expression levels, which is further enhanced by the absence of transgene silencing in the chloroplast. The chloroplast genome has thus become an ideal bioengineering target to produce high-value commodities, such as vitamins, vaccines, as well as medical and agricultural compounds, at large scales (Verma et al., 2008; Maliga and Bock, 2011; Jin and Daniell, 2015; Adem et al., 2017).

Chloroplast transformation and the creation of transplastomic germplasm are expected to add to the repertoire of technologies available for agriculture and industry. Several methods have been reported for chloroplast transformation in various plant species, such as polyethylene glycol (PEG) treatment of protoplasts or biolistic bombardment of leaves, suspension cell cultures, and embryogenic callus tissue (Golds et al., 1993; Svab and Maliga, 1993; Bock, 2015). However, chloroplast transformation efficiency is low and a simple and efficient method that can be applied to various plant species would enable broad application of this technology.

Cell-penetrating peptides (CPP) are used as carriers to deliver proteins, nucleotides, and small drug molecules in a spatially and temporally controlled manner inside cells, tissues, and organs to treat various human diseases (Guidotti et al., 2017; Borrelli et al., 2018). To date, various types of CPPs have been developed, including synthetic CPPs identified by screening peptide libraries (Guidotti et al., 2017; Borrelli et al., 2018), and used in mammalian cells and in plant cells (Mae et al., 2005; Chugh and Eudes, 2008; Lakshmanan et al., 2013; Numata et al., 2018). The mechanism by which CPPs are transported into cells has been studied in mammal cells in the context of their use in drug delivery (Guidotti et al., 2017; Borrelli et al., 2018). Although CPPs take different routes to enter cells (Guidotti et al., 2017), most CPPs are thought to enter the cell indirectly *via* clathrin-dependent or -independent endocytosis, macropinocytosis, or phagocytosis rather than *via* direct penetration through the plasma membrane (Mayor and Pagano, 2007; Madani et al., 2011; Guidotti et al., 2017; Borrelli et al., 2018).

We previously designed a chimeric peptide containing a CPP, nine lysine-histidine (KH) repeats for DNA binding, and a short chloroplast-transit peptide (CTP; Yoshizumi et al., 2018). The polycationic nature of the CPP allows its passage across cellular membranes and condenses negatively charged DNA with its constituent lysines, while histidines are critical for the release of the DNA into the endosomal vesicles (Chen et al., 2000; Leng et al., 2007; Lakshmanan et al., 2013). Using a complex between the peptide and plasmid DNA (peptide–DNA complex), we delivered DNA to the chloroplast and showed integration of the exogenous genes harbored by the plasmid into the chloroplast genome of mesophyll cells from *Arabidopsis* and tobacco (*Nicotiana tabacum*; Yoshizumi et al., 2018). Peptide–DNA complexes targeted to the chloroplast can integrate into the chloroplast genome with high selectivity (Yoshizumi et al., 2018). We recently achieved successful transient transformation of various plastid types using a complex between plasmid DNA and two peptides, a CTP (for plastid targeting) and a CPP (for cell penetration), resulting in a DNA–CTP–CPP complex (Thagun et al., 2019). However, how and when the peptide–DNA complex enters mesophyll cells by crossing the cell wall and the plasma membrane before reaching the chloroplast is unknown.

In the present study, we investigated how the peptide–DNA complex is transported to the chloroplast stroma after infiltration of the leaf surface by tagging the complex with fluorescent probes and visualizing it by confocal laser scanning microscopy (CLSM). We detected the penetration of the peptide–DNA complex into chloroplasts within 6h, showing that it had passed through both the chloroplast outer and inner membranes. We observed that the peptide–DNA complex went through three phases during penetration into the chloroplast. Our findings thus reveal the behavior of the peptide–DNA complex during chloroplast transformation of plant cells.

## Materials and Methods

### Plant Materials and Growth Conditions

*Arabidopsis* (*Arabidopsis thaliana*, At) Columbia accession (Col-0) was used for all experiments. A transgenic line expressing *CHUP1-NT-GFP* was selected among T3 lines with normal chloroplast positioning, based on green fluorescent protein (GFP) fluorescence around the chloroplast outer envelope (Oikawa et al., 2008). The line was used to examine the delivery of the KH-AtOEP34–DNA complex (cpPD complex) to the chloroplast outer membrane and stroma. For plasmid DNA delivery and integration of exogenous DNA into chloroplasts, plants were grown for 2months under a 8-h light/16-h dark short-day photoperiod on soil to gain a large leaf.

### Peptide and Plasmid Preparation

KH-AtOEP34 [protein sequence: (KH)_9_-MFAFQYLLVM] was previously synthesized and purified (Yoshizumi et al., 2018). The purity and the molecular weight of the peptide were confirmed using high-performance liquid chromatography with an InertSustain C18 column (GL Science, Tokyo, Japan) and through matrix-assisted laser desorption/ionization-time-of-flight mass spectrometry (Yoshizumi et al., 2018), respectively. We have previously constructed the plasmid *PpsbA*-*aadA-sGFP*-*TpsbA* (Yoshizumi et al., 2018). In this study, we used the plasmid for DNA delivery as part of the complex with the peptide KH-AtOEP34. DNA fragments for the *psbA* promoter (PpsbA) and *psbA* terminator (TpsbA) were amplified with the primer sets (5'-GAAGATCTGCAAGAAAATAACCTCTCCTTC-3' and 5'-CTCCTCGCCCTTGCTCACCATTCTCTCTAAAATTGCAGTCATGGTAAAATCTTGG-3') for *psbA* promoter and (5'-GAAAAATTCTATAGAAACTTCTCTCAATTAGGATCCTGGCCTAGTCTATAGG-3' and 5'-GAAGATCTGCAAGAAAATAACCTCTCCTTC-3') for *psbA* terminator. The DNA fragment *PpsbA:sGFP:TpsbA* was generated by combining each PCR fragment and cloned into the pUC19 vector (Yoshizumi et al., 2018). We also used the plasmid *Prrn-aadA-sfGFP-Trps* for integrating exogenous DNA into *Arabidopsis* plastid genome for GFP expression analysis. The plasmid was constructed following the methods (Lutz et al., 2007). *Prrn* gene is a sequence for promoter of plastidic ribosomal RNA, *aadA-sfGFP* gene is a sequence coding aminoglycoside adenyltransferase fused to sfGFP, and *Trps* is a terminator sequence of plastidic ribosomal RNA 16S. In the plasmid, 5' and 3' flanking regions of the *Prrn-aadA-sfGFP-Trps* region contains DNA sequence for transfer RNA L (*trnl*) and transfer RNA A (*trnA*) for recombination with chloroplast genome, respectively. The plasmids used in this study are listed in Supplementary Table S1.

To construct KH-AtOEP34–DNA complexes in a 0.5N/P ratio (with N being the number of amine groups from the peptide and P being the number of phosphate groups from the plasmid DNA), plasmid DNA was purified with the Qiagen DNA extraction kit. After an incubation of 1min at 23°C and thorough mixing, the KH-AtOEP34 peptide was added to the DNA solution and incubated for 1h at 23°C (Midorikawa et al., 2019; Miyamoto et al., 2019).

To visualize KH-AtOEP34–DNA complex, the plasmid DNA was labeled with cyanine fluorescent dye (Cy3) using a Nucleic Acid Labeling Kit (Label IT tracker Reagent, Mirus). After purification of the plasmid DNA using ethanol precipitation, 5μg of plasmid DNA conjugated to Cy3 in 100μl was mixed with KH-AtOEP34 at an N/P ratio of 0.5 (Chuah et al., 2015; Chuah and Numata, 2018).

### Dynamic Light Scattering Measurement

The peptide–DNA complex were characterized with a zeta potentiometer (Zetasizer Nano-ZS; Malvern Instruments, Ltd., Worcestershire, United Kingdom) following published methods (Chuah et al., 2015). Each mixture of cpPD complex was prepared to a final volume of 800μl using ultrapure water (Milli-Q) at an N/P ratio of 0.5. The mixture was immediately placed into a cuvette to measure the zeta potential and the size with a zeta potentiometer, and the averaged data were obtained using Zetasizer software version 6.20 (Malvern Instruments, Ltd.).

### Infiltration of Peptide–DNA Complexes

Around 100μl of peptide–DNA complex solution was infiltrated with a syringe into the abaxial side of a leaf of a 2-month-old *Arabidopsis* plant grown in short-day conditions (Chuah et al., 2015). The leaves were then cut into four pieces and observed by CLSM at different times after infiltration.

### Confocal Laser Scanning Microscopy Analysis

Fluorescence signals from the peptide–plasmid DNA complex labeled with Cy3 (cpPD-Cy3 complex) or GFP localized at the chloroplast outer membrane were detected using the CLSM (Zeiss LSM880) with a 63x oil immersion objective (Plan-Apochromat 63x/1.4 Oil DIC M27) and a zoom (x 1.5–2.5) in Airy scan mode (Carl Zeiss, Jena, Germany) with an Argon and DPSS laser for excitation of GFP and Cy3 and the following excitation/emission wavelengths: 488/495–550 nm for GFP and 561/495-620 nm for Cy3. Time-lapse images were taken every 0.5–5s for 120–600s and stacked as a movie file using Imaris (Carl Zeiss, Jena, Germany) or Image J (Fiji; Schindelin et al., 2012) after taking single images from the surface to bottom of the mesophyll cell. The plasma membrane of leaf sections was stained with 20μM FM4-64 dye (Thermo Fisher, United States) for 10min after infiltration of the cpPD-Cy3 complex for 1–12h. The leaf sections were then rinsed in distilled water once before observation by confocal microscopy using excitation/emission wavelengths: 561/565–590nm for Cy3, 561/620–650nm for FM4-64 dye, and 488/660–700nm for chlorophyll autofluorescence. Chloroplast DNA was stained with SYBR Green I (Dragan et al., 2012) for 30min after infiltration of the cpPD-Cy3 complex for 6h. SYBR Green I fluorescence was observed by confocal microscopy using excitation/emission wavelengths: 488/490–540nm for SYBR Green I, 488/660–720nm for chlorophyll autofluorescence, and 561/565–590nm for Cy3.

### Field Emission-Scanning Electron Microscopy Analysis

Field emission-scanning electron microscopy (FE-SEM; Gemini 3000, Zeiss, Germany) was performed following a previous method (Sogawa et al., 2020). A sample of the peptide–DNA complex (cpPD complex) was prepared as described above. The cpPD complexes were enveloped in 2% (w/v) agarose and sectioned to 1–2mm for FE-SEM observation. Leaf sections treated with the cpPD complex were embedded in epoxy resin and sectioned to 100μm for FE-SEM observation.

### Statistical Analysis

The method followed to count the number of peptide–plasmid DNA complexes labeled with Cy3 (PD-Cy3 complex) inside and outside leaf mesophyll cells is described in Supplementary Figure 1. The ratio of cpPD-Cy3 inside and outside cells was calculated. The frequency of the Phase I-IV was calculated as each number of the Phase I-IV chloroplasts divided by the total number of the chloroplasts in the cell, which has the chloroplasts stained with SYBR Green I with the cpPD-Cy3 complexes. The frequency of the cpPD-Cy3 complexes bound to the cp-nucleoids was calculated as divided by the total number of the cpPD-Cy3 complexes on the chloroplast.

## Results

### Characterization of the Peptide–DNA Complex Used for Plasmid DNA Delivery to the Chloroplast and Exogenous DNA Integration Into the Chloroplast Genome

**Figure 1:**
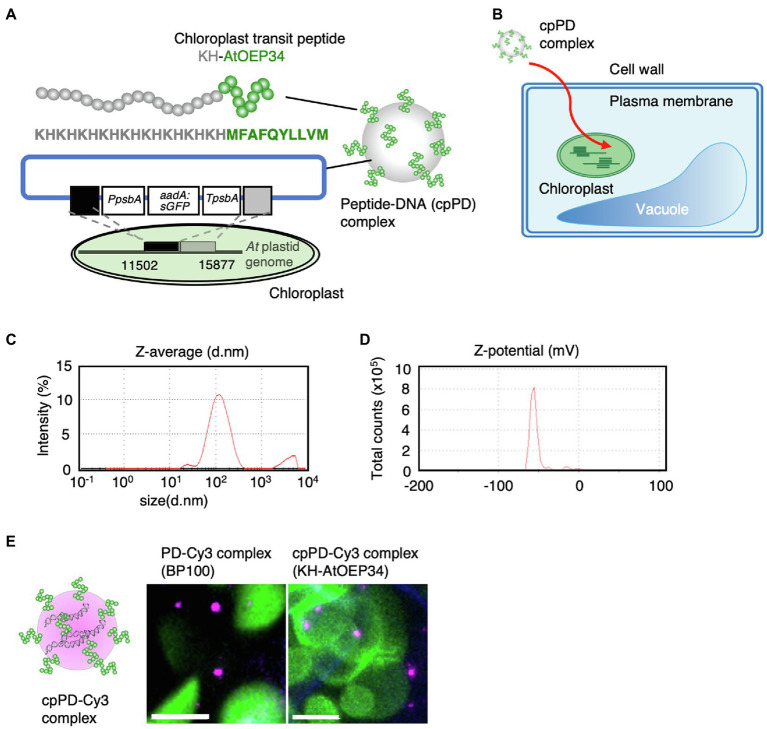
Characterization of the peptide–DNA complex used in this study. **(A)** Schematic diagram of the peptide–DNA complex KH-AtOEP34–pDNA (cpPD complex) used in this study. The protein sequence of the KH-AtOEP34 peptide is shown in gray (KH) or green (AtOEP34). Plasmid DNA used for integrating exogenous DNA into *Arabidopsis thaliana* (*At*) plastid genome is shown below. Each box represents DNA fragments, *PpsbA*, promoter of psbA; *aadA-sGFP*, a sequence coding aminoglycoside adenyltransferase (*aadA*) fused to *sGFP*; *TpsbA*, terminator sequence of *psbA*; black and gray boxes; sequence used for integration into chloroplast genome. **(B)** Schematic diagram of the path of cpPD complexes from the extracellular space of a leaf cell to the chloroplast stroma. **(C,D)** Z-average **(C)** and Z-potential **(D)** of cpPD complexes in solution. **(E)** Left, schematic diagram of the KH-AtOEP34–pDNA complex labeled with Cy3 (cpPD-Cy3 complex). Right, representative images of PD-Cy3 complexes (BP100) and cpPD-Cy3 complexes in leaf mesophyll cells. Scale bars denote 10μm.

To understand how the peptide-pDNA complex is internalized into a cell and delivered to chloroplast nucleoids, we tracked the peptide-pDNA complex using imaging technology with the previously designed peptide, KH-AtOEP34, and the plasmid *PpsbA*-*aadA-sGFP*-*TpsbA* (Yoshizumi et al., 2018). The chimeric peptide KH-AtOEP34 consists of a DNA-binding cationic peptide and a signal peptide for targeting to the chloroplast outer membrane, in complex with the plasmid to assess gene delivery to the chloroplast (Figure 1A; Yoshizumi et al., 2018). The negative charge of the plasmid DNA allowed its complexation with the cationic peptide (Figure 1A). The successful delivery of these peptide–DNA (cpPD) complex entails the penetration of the cell wall and plasma membrane, followed by delivery to the chloroplast and DNA integration into the chloroplast genome, as shown in Figure 1B.

We first determined the hydrodynamic size and surface charge (Z-potential) of the cpPD complexes, following a method that differed slightly from the one reported previously (Yoshizumi et al., 2018) to stabilize the size of the cpPD complexes (Midorikawa et al., 2019; Miyamoto et al., 2019). Dynamic light scattering (DLS) analysis revealed that more than 90% of the cpPD complexes are approximately 130nm in diameter when in solution (Figure 1C) with a Z-potential of −55mV (Figure 1D). Therefore, the size of these cpPD complexes was three times smaller than in our earlier publication and their Z-potential was halved compared to complexes generated using our previous method (Yoshizumi et al., 2018).

To track the progress of cpPD complexes inside cells, we labeled the plasmid DNA with the fluorescent dye Cy3 and detected the resulting cpPD-Cy3 complexes inside the cell by CLSM (Figure 1E). The cpPD-Cy3 complexes were found as particles inside *Arabidopsis* cells (Figure 1E). We hypothesize that the larger cpPD-Cy3 complex *in planta* relative to that obtained by DLS may reflect the spatial resolution limits of CLSM.

**Figure 2:**
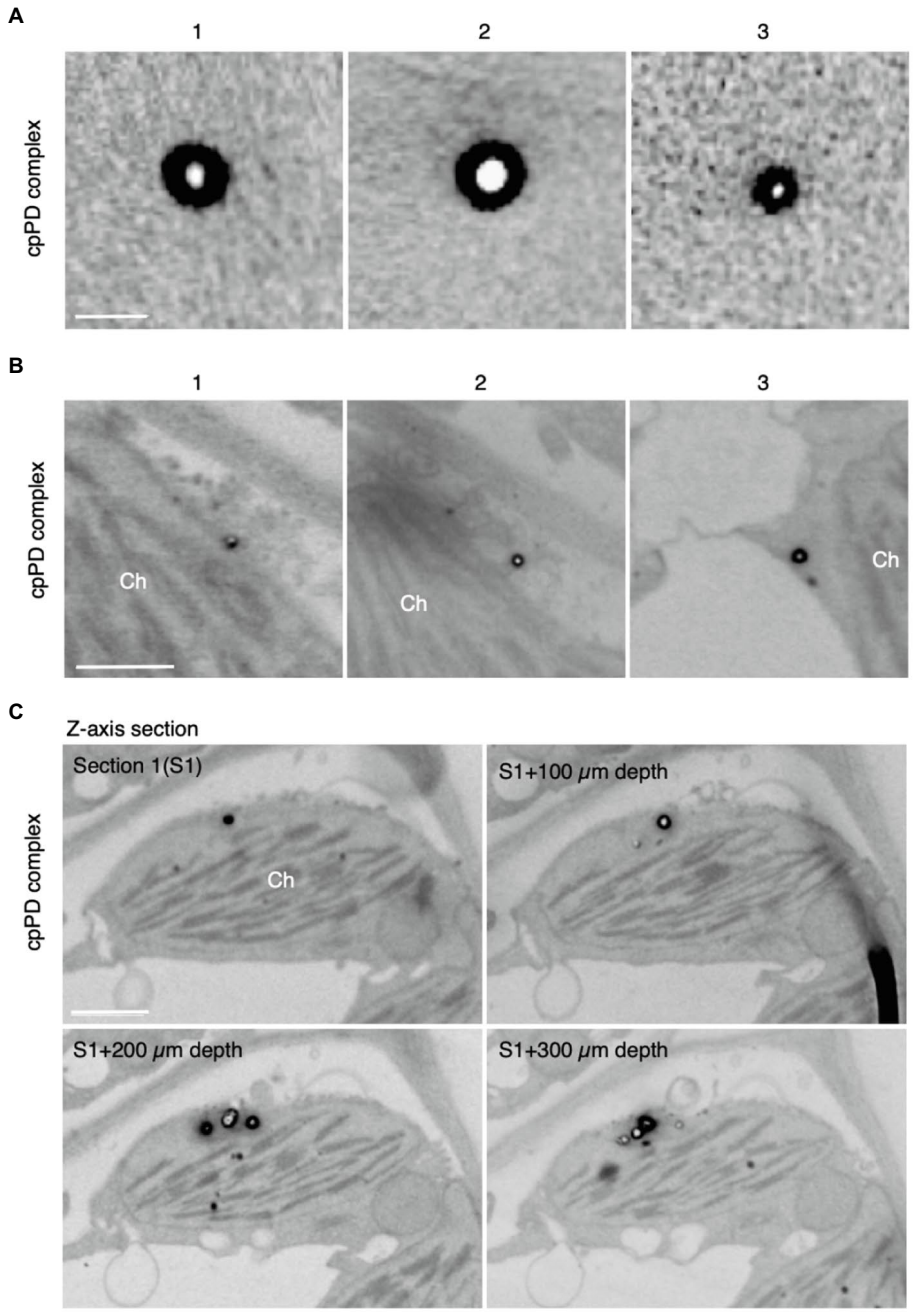
Field emission-scanning electron microscopy (FE-SEM) analysis of KH-AtOEP34-pDNA complexes. **(A,B)** Three different images of the KH-AtOEP34–pDNA (cpPD complexes; 1–3) in agarose gel **(A)** and around chloroplasts (Ch; **B**) are shown. **(C)** Four consecutive *z*-axis sections showing cpPD complexes on the chloroplast surface. The sections are cut at every 100 μm depth. Bars=100 nm in **(A)** and 1μm in **(B,C)**.

Next, we characterized cpPD complexes by FE-SEM. When embedded in 2% agarose gel, cpPD complexes appeared as white globular structures with a black circumference (Figure 2A). To confirm these characteristics *in vivo*, we infiltrated a solution of cpPD complexes into *Arabidopsis* leaves and visualized the cpPD complexes in the cells by FE-SEM. We observed a similar appearance of the cpPD complexes *in vivo* to that seen *in vitro* (Figure 2B). Some of the cpPD complexes were located close to chloroplasts (Figure 2C). With the higher resolution of SEM, the diameter of the cpPD complexes was about 100nm (Figure 2). These results suggest that the cpPD complexes maintain their shape and size when infiltrated into living plant cells.

### Peptide–DNA Complexes Are Delivered to the Chloroplast Surface After Passing Through the Cell Wall and the Plasma Membrane

**Figure 3:**
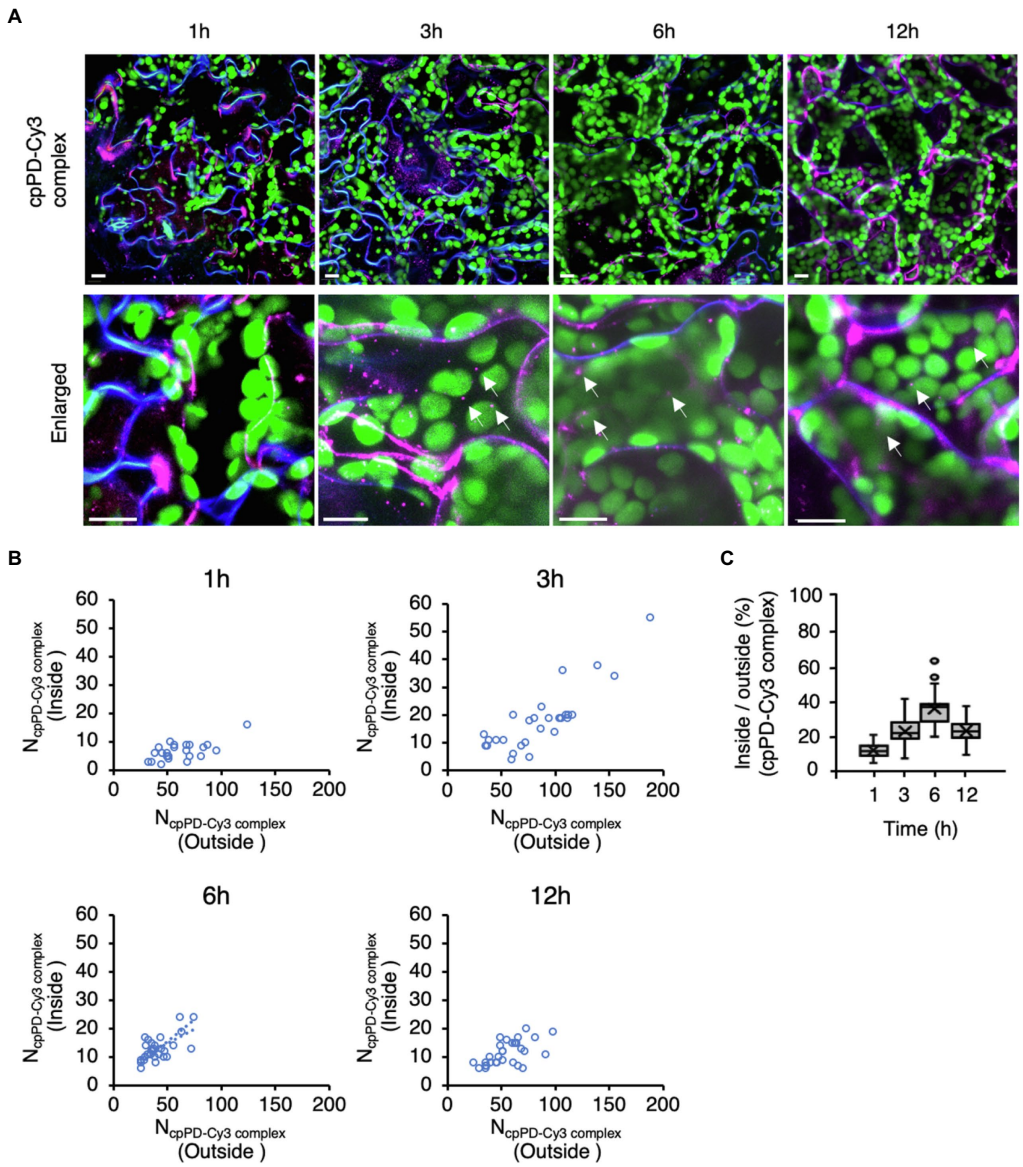
Time-dependent penetration of KH-AtOEP34–pDNA complexes into leaf mesophyll cells. **(A)** Subcellular localization of KH-AtOEP34–pDNA complexes labeled with Cy3 (cpPD-Cy3; magenta) in *Arabidopsis* leaf mesophyll cells at 1, 3, 6, and 12h after infiltration. Enlarged images are shown in the lower panels. The plasma membrane (blue) is stained with FM4-64. Chloroplasts (green) are visualized as chlorophyll autofluorescence. Scale bars denote 10μm. **(B)** Scatterplots showing the relative spatial distribution of cpPD-Cy3 complexes at the different time points shown in **(A)**. Each circle represents a cell with the number of cpPD-Cy3 complexes outside (*x*-axis), N_cpPD-Cy3 complex_ (Outside), and inside (*y*-axis), N_cpPD-Cy3 complex_ (Inside), the cell (*n*>24). **(C)** Ratio of the number of cpPD-Cy3 complexes inside and outside cells shown in **(B)**, as box plots. The horizontal lines represent the mean and the cross represents the median. Circles represent outliers.

To understand how cpPD complexes penetrate into the cell after infiltration from the leaf surface, we tracked the progress of cpPD-Cy3 complexes in *Arabidopsis* mesophyll cells (Figure 3). We stained the plasma membrane with the dye FM4-64 (Rigal et al., 2015) to visualize the cell boundary at 1, 3, 6, and 12h after infiltration (Figure 3A) and generated a full *z*-projection from *z*-stack images with the help of ImageJ (Schindelin et al., 2012; Supplementary Figure 1). The images revealed that the cpPD-Cy3 complexes gradually penetrate into leaf mesophyll cells in a time-dependent manner (Figure 3A).

To clarify how the cpPD-Cy3 complexes penetrate into leaf mesophyll cells, we scored the number of complexes accumulating outside and inside the cell as a function of time since infiltration (Figure 3B). The number of cpPD-Cy3 complexes inside the cell was the lowest after 1h but increased until 6h (Figure 3B), as evidenced by the gradual rise of the average ratio between the complex numbers inside and outside the cell from about 10% at 1h, 20% at 3h, and reaching 35% at 6h (Figure 3C). However, longer incubation times resulted in a decrease of this ratio to about 20% at 12h (Figure 3C). We examined the effect of wortmannin and brefeldin A (BFA), which inhibit endocytosis and autophagy, on the cpPD-Cy3 complexes penetration into the cell (Supplementary Figure 2). The result showed that degradation of the cpPD-Cy3 complexes was suppressed at 12h. These results suggested that cpPD-Cy3 complexes penetrate into the intercellular space of the leaf through stomata within 1h and gradually invade the cell from 3 to 6h before delivering their DNA cargo to the chloroplast, and degrade in vacuole at 12h. Based on the above results, we focused our observations of cpPD-Cy3 complexes penetrating chloroplasts at 6h after infiltration.

### cpPD Complexes Are Surrounded by the Chloroplast Outer Membrane for Their Internalization

**Figure 4:**
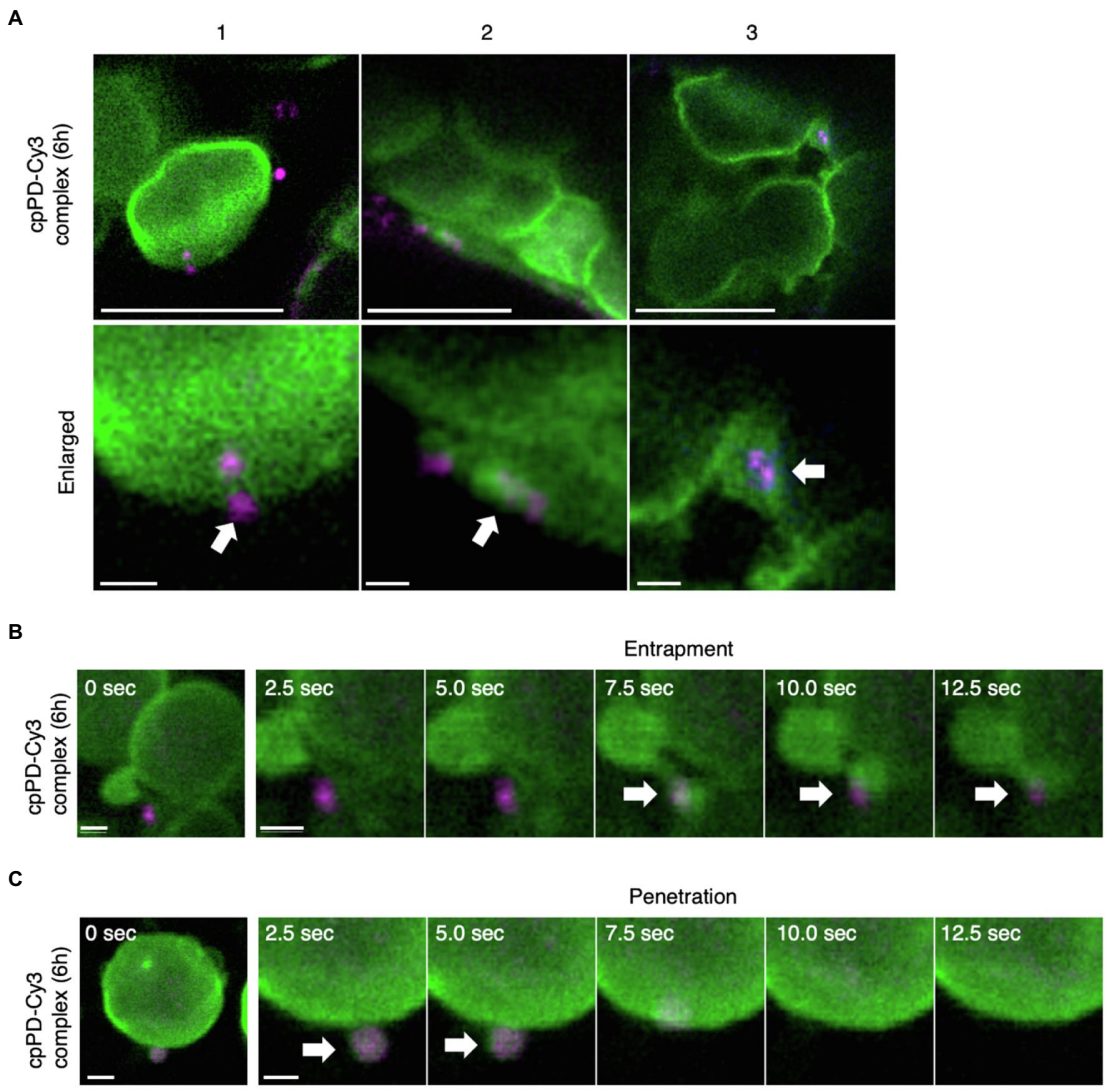
KH-AtOEP34–pDNA complexes localize to the chloroplast outer membrane. **(A)** Three representative examples (1–3) of KH-AtOEP34–pDNA localization patterns, as visualized by Cy3 fluorescence (cpPD-Cy3; magenta) around chloroplasts (green). Bars=5μm. Enlarged images in the lower panels represent cpPD-Cy3 complexes associated with (1), being enveloped by (2), or integrated into (3), the chloroplast outer membrane (green). Arrows denote cpPD-Cy3 complexes. Bars=1μm. **(B,C)** Time-lapse images illustrating entrapment **(B)** and incorporation **(C)** of cpPD-Cy3 complexes by the chloroplast outer membrane.

We showed previously that cpPD-Cy3 complexes are trapped on the chloroplast outer membrane and become gradually internalized into the organelle (Yoshizumi et al., 2018). To further determine how cpPD-Cy3 complexes enter the chloroplast, we carefully observed individual cpPD-Cy3 complexes near chloroplasts whose outer membranes were fluorescently-labeled by a fusion protein between the N terminus of CHLOROPLAST UNUSUAL POSITIONING1 (CHUP1) and the GFP. Consistent with our previous results, cpPD-Cy3 complexes showed an association with the chloroplast outer membrane (Figure 4A-1), followed by the attachment of the complexes to a membrane protrusion formed on the chloroplast surface (Figure 4A-2), culminating in complexes being enveloped by the protrusion, as would be observed for phagocytosis (Figure 4A-3).

To ascertain that individual cpPD-Cy3 complexes are internalized into the chloroplast, we then performed time-lapse imaging by CLSM. After cpPD-Cy3 complexes were trapped by a chloroplast protrusion (Figure 4B; Supplementary Movie 1), they gradually appeared to be integrated into the chloroplast before becoming undetectable (Figure 4C, white arrows; Supplementary Movie 1). These results confirmed that cpPD-Cy3 complexes are recognized by the chloroplast outer envelope and then internalized into the organelle.

### cpPD Complexes Accumulate at the Around Chloroplast Nucleoids

**Figure 5:**
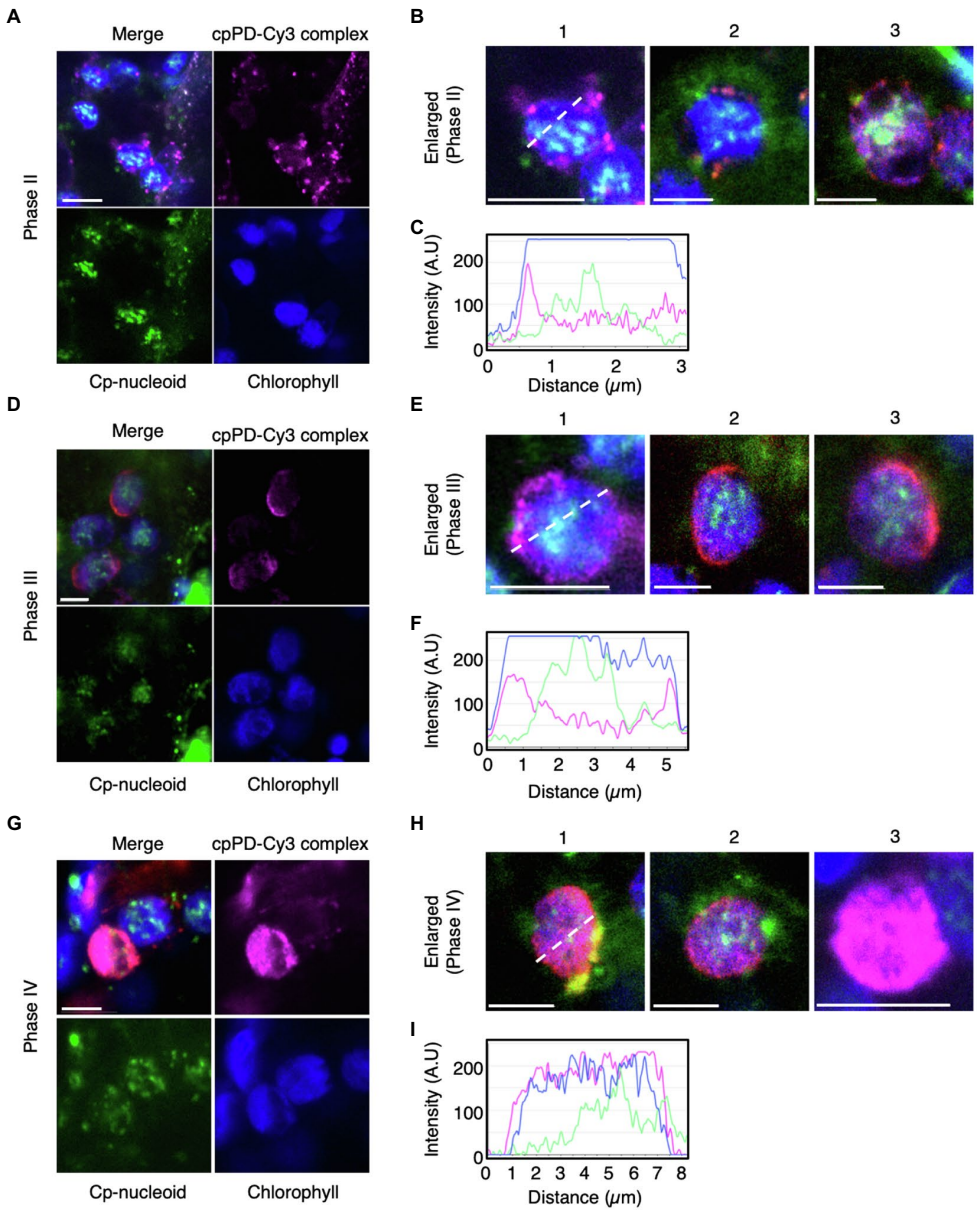
Three different phases of cpPD-Cy3 complexes localization around the chloroplast surface. **(A,B,D,E,G,H)** KH-AtOEP34–pDNA complexes labeled with Cy3 (cpPD-Cy3; magenta) localize adjacent to the chloroplast surface (blue). The chloroplast genome (cp-nucleoid) was visualized with SYBR Green I (green). Localization of cpPD-Cy3 complexes as dots **(A,B)**, stacks **(D,E)** around the chloroplast surface, and throughout the chloroplast stroma **(G,H)**. **(B,E,H)** Enlarged images of cpPD-Cy3 complexes from Phases II–IV based on the accumulation patterns of cpPD-Cy3 complexes defined in **(A,D,G)** in **(B,E,H)** as follows: the 1-3 means different chloroplasts.. **(C,F,I)** Fluorescence density profiles along the white dashed line for cpPD-Cy3 complexes at Phase I–III. Bars=5μm.

**Figure 6:**
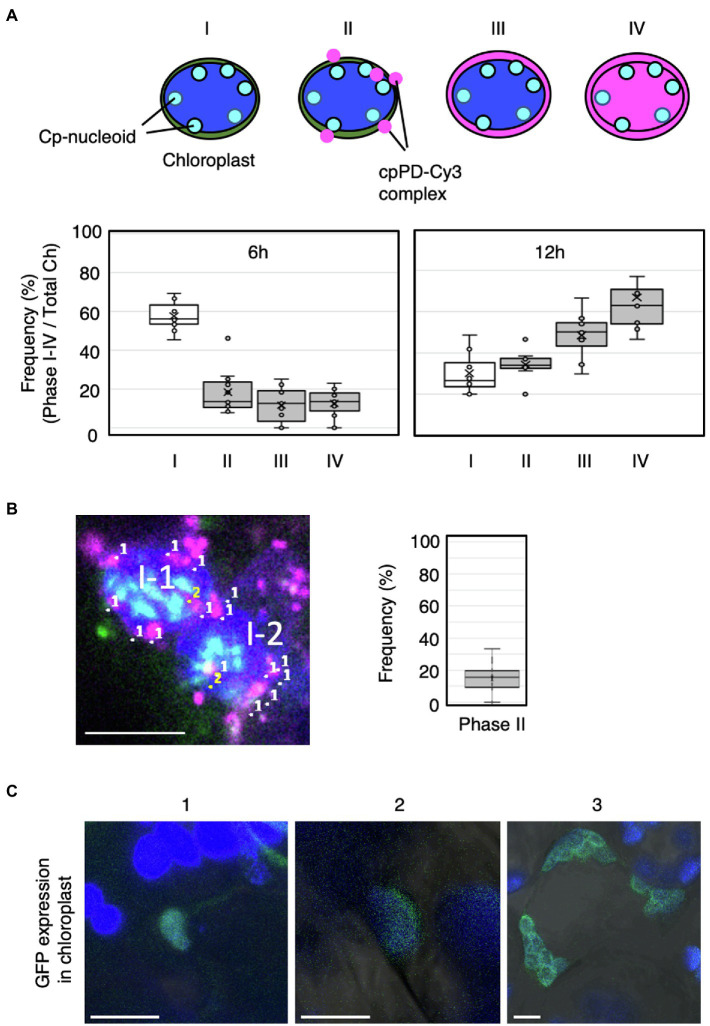
Frequency of cpPD-Cy3 complex accumulation in and around the chloroplast. **(A)** Top, schematic representation of the four phases of cpPD-Cy3 complexes penetrating into chloroplasts. Bottom, distribution of the frequency of chloroplasts showing Phase I–IV (I–IV) stages at 6 and 12h after infiltration. **(B)** Numbers in the image at the left panel represent cpPD-Cy3 complexes (magenta) (1) and the complex with nucleoids (sky blue) (2) in two mesophyll cells (II-1, II-2). The frequency of cpPD-Cy3 complexes bound to the cp-nucleoids is shown. **(C)** Three different images (1–3) of sfGFP (green) accumulation in transformed chloroplasts by the cpPD complex. Chloroplasts (blue) are visualized as chlorophyll autofluorescence. Bars=5μm.

To test whether cpPD-Cy3 complexes reach chloroplast nucleoids (cp-nucleoids; Powikrowska et al., 2014), we stained chloroplast DNA with SYBR Green I (Dragan et al., 2012) and visualized cp-nucleoids and cpPD-Cy3 complexes simultaneously at 6 and 12h after infiltration. The cp-nucleoids appeared as green foci inside chloroplasts, which were detected based on chlorophyll autofluorescence (shown in blue). Importantly, we observed the co-localization of several cpPD-Cy3 complexes (shown in magenta) and cp-nucleoids (Figures 5A,D,G). We classified the appearance of cpPD-Cy3 complexes and adjacent cp-nucleoids into four stages, namely Phase I (a control: chloroplast without the cpPD-Cy3 complexes) and Phases II–IV (Figures 5, 6). Phase II is characterized by the binding of cpPD-Cy3 complexes to the chloroplast surface, with only a few complexes being close to cp-nucleoids (Figures 5A–C). Phase III was associated with many cpPD-Cy3 complexes gathering along the chloroplast surface (Figures 5D–F). Finally, the strong accumulation of DNA molecules, labeled with Cy3 and released from the cpPD-Cy3 complexes, inside the chloroplast, constituted Phase IV (Figures 5G–I). From the images shown in Figures 5A,D,G, we selected individual representative chloroplasts (Figures 5B,E,H) to measure their fluorescence intensity profiles along a line transecting the organelle. The fluorescence profiles of Phase I-IV clearly showed different localization patterns of cpPD-Cy3 complexes and indicated the gradual co-localization of plasmid DNA with cp-nucleoids (Figures 5C,F,I).

To better characterize how plasmid DNA moves from the chloroplast outer membrane to cp-nucleoids, we scored the number of chloroplasts presenting Phases I–IV at 6 and 12h after infiltration (Figure 6A). After 6h, most chloroplasts lacked detectable cpPD complexes around their periphery, while chloroplast with cpPD-Cy3 complexes were broadly evenly distributed across all Phases (Figure 6A). By 12h after infiltration, far fewer chloroplasts were devoid of cpPD-Cy3 complexes nearby, with Phases II–IV demonstrating a steep rise over 6h. In addition, Phase III and Phase IV stages were more frequent after 12h than the Phase II stage, with ~60% of chloroplasts exhibiting a Phase IV stage (Figure 6A). Based on these results, Phase I–IV stages clearly reflected the time-dependent steps followed by cpPD-Cy3 complexes accumulating inside chloroplasts (Figures 5, 6).

To determine if cpPD-Cy3 complexes came in close proximity to cp-nucleoids, we next scored chloroplasts for co-localization between the fluorescence signals derived from Cy3 (cpPD-Cy3 complexes) and SYBR Green I (cp-nucleoids): about 15% of cpPD-Cy3 complexes did colocalize with cp-nucleoids; this proximity would likely allow the incorporation of the plasmid DNA delivered by the complexes into the chloroplast genome (Figure 6B).

As a final test for the effective incorporation of plasmid DNA, *Prrn-aadA-sfGFP-Trps*, into the chloroplast genome, we attempted to detect fluorescence from a transgene encoding the superfolder GFP (sfGFP; Pedelacq et al., 2006; Fujii and Kodama, 2015). GFP fluorescence in a portion of chloroplast (Figure 6C-1) and most chloroplasts (Figures 6C-2,3) in a cell was observed. These results suggested that the plasmid DNA was incorporated inside the chloroplast stroma and integrated into the chloroplast genome. Based on our observations, the cpPD complexes were clearly internalized into the chloroplast *via* engulfment by the chloroplast outer envelope.

## Discussion

**Figure 7:**
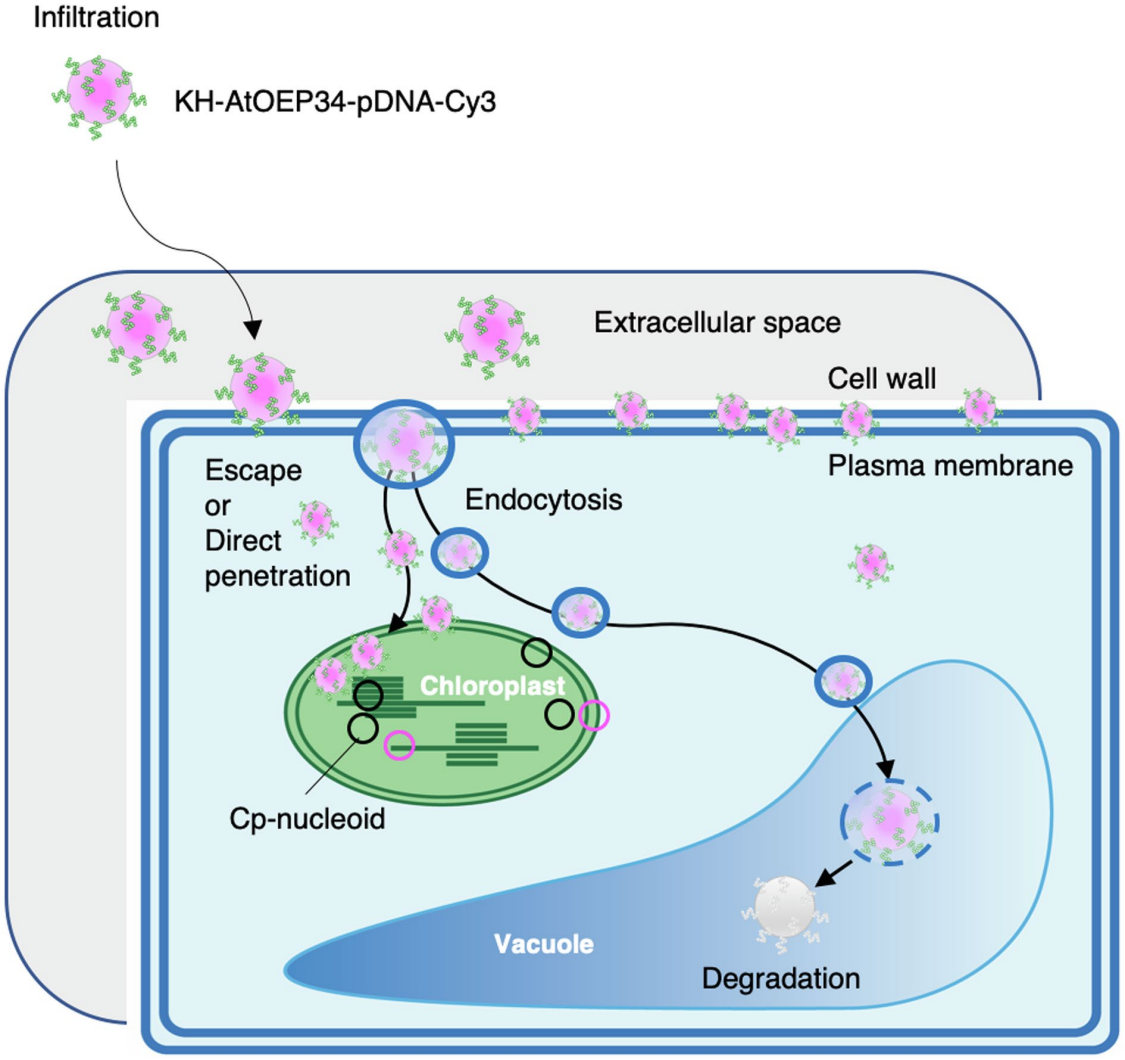
Proposed model for the penetration of KH-AtOEP34–pDNA complexes from outside the cell to the chloroplast stroma. After infiltration of KH-AtOEP34–pDNA complexes labeled with Cy3 (cpPD-Cy3 complex) at the leaf surface, cpPD-Cy3 complexes move to the leaf extracellular space. Then, cpPD-Cy3 complexes penetrate into the cell *via* endocytosis or direct penetration; however, some complexes are trapped by the cell wall and/or the plasma membrane. In the cytosol, cpPD-Cy3 complexes are either targeted to the vacuole for degradation by endocytosis and autophagy, or escape from the degradation and become incorporated into the chloroplast.

In this study, we employed peptide–DNA complexes formed by complexation of the KH-AtOEP34 peptide to a plasmid of interest to efficiently deliver DNA to the chloroplast. The size of these cpPD complexes was about 130nm, as determined by DLS analysis (Figure 1). Furthermore, these complexes localized near the chloroplast, as demonstrated by FE-SEM observations (Figure 2). Confocal microscopy analysis and the generation of full *z*-stack images revealed that cpPD-Cy3 complexes penetrate into cells and chloroplasts within 6h after their infiltration into the extracellular space. Inhibitor of endocytosis and autophagy suppressed the decrease of the cpPD-Cy3 at 12h, suggesting that cpPD-Cy3 complexes keep direct penetration and suppress degradation in vacuole within 12h (Figure 3; Supplementary Figure 2). Moreover, we documented the internalization of cpPD-Cy3 complexes into the chloroplast *via* trapping by the chloroplast outer membrane, which eventually brought a fraction of cpPD-Cy3 complexes adjacent to cp-nucleoids. Based on our findings, we propose a cascade model for the transport of cpPD-Cy3 complexes from outside the cell to inside the chloroplast stroma (Figure 7). The cpPD-Cy3 complexes infiltrated from the leaf surface likely penetrate inside the cell by using endocytosis or direct penetration through stoma, reaching the extracellular space, then the cell wall, and finally the plasma membrane (Figure 7). At this stage, a pool of cpPD-Cy3 complexes escaping from endocytosis and autophagy, which lead to degradation in vacuole, will be delivered to the chloroplast surface, where they become internalized into the chloroplast *via* an unknown mechanism involving chloroplast membrane flexibility (Figure 7).

Cellular uptake of cpPD-Cy3 complexes proceeded from the extracellular space to the chloroplast in an orderly and timely manner, reaching a peak at 6h after infiltration (Figure 3). The uptake of fluorescein-isothiocyanate (FITC)-labeled BP100 peptides into tobacco BY-2 cells did not attain 50% of maximum levels after a 6-h incubation and only reached saturation after 12h (Eggenberger et al., 2011). Our group previously explored the transfection efficiency of other CPPs, such as R_9_-BP100, (KH)_9_-BP100, and R_9_-Tat2, complexed to plasmid DNA harboring a *Renilla luciferase* (*RLuc*) transgene (Lakshmanan et al., 2013; Numata et al., 2014). When these various complexes were infiltrated into tobacco and *Arabidopsis* leaves, (KH)_9_-BP100, R_9_-BP100, and R_9_-Tat2 exhibited the highest transfection efficiency after 12h, although R_9_-BP100 and R_9_-Tat2 were faster than (KH)_9_-BP100 (Lakshmanan et al., 2013). High transfection efficiencies into *Arabidopsis* leaves were also recently achieved with the BPCH7 peptide, resulting from the conjugation of the synthetic peptides BP and CH7. BPCH7 is an efficient cell-penetrating peptide with a circular DNA-binding domain that allows the release of the associated DNA cargo into the cell (Chuah and Numata, 2018). The intracellular trafficking of BPCH7–pDNA complexes revealed the even distribution of the complexes throughout the cell at 6h after infiltration (Chuah and Numata, 2018). Compared to these CPPs, the cellular uptake of the cpPD complex described here is slightly lower but nevertheless sufficient to penetrate through the cell wall and plasma membrane. Although, the pattern of time-dependent penetration of cpPD-Cy3 complexes was similar to that of (KH)_9_-BP100–pDNA-Cy3 complexes (Figure 3C; Supplementary Figure 3), the cellular uptake efficiency of cpPD-Cy3 complexes remained lower than that of PD-Cy3 complexes (Supplementary Figure 4). This variation in uptake efficiency may be related to the secondary structure and of amino acid charge of the CPP, which will affect cellular uptake mechanisms like endocytosis and pinocytosis (Mayor and Pagano, 2007; Madani et al., 2011; Guidotti et al., 2017; Borrelli et al., 2018). Indeed, we found that vesicles encircle the cpPD-Cy3 complexes and stayed close to the chloroplast during time-lapse analysis (Supplementary Movie 2).

Because many cpPD-Cy3 complexes appeared trapped outside the cell periphery, as revealed by staining with FM-4-64 or calcofluor even 12h after infiltration (Figure 3A), we consider that most cpPD-Cy3 complexes remain in the extracellular space, including the cell wall (Figure 3A). The cell wall consists of carbohydrates and phenolic compounds with a few structural proteins that together orchestrate the complex plant architecture and changes in response to growth stages (Hofte and Voxeur, 2017). We recently characterized the structure of the cell wall in BY-2 cells at nanometer resolution using high-speed atomic force microscopy (Yilmaz et al., 2020). We observed aligned as well as disordered cellulose fibrils coexisting in the outermost layer of the cell wall (Yilmaz et al., 2020). This complex structure suggests that penetration of cpPD-Cy3 complexes into cells should be blocked by the organized structure and/or chemical components of the cell wall. Our next challenge will be to overcome these cell wall properties for efficient penetration of peptide-DNA complexes.

After penetration into the cell, cpPD-Cy3 complexes were delivered and internalized into the chloroplast by their constituent signal peptide from the chloroplast outer membrane protein OEP34 (Yoshizumi et al., 2018). However, how the cpPD-Cy3 complexes then cross both the chloroplast outer and inner membranes to arrive in the chloroplast stroma was unclear. In the present study, we discovered that some of the cpPD-Cy3 complexes are trapped by protrusions of the chloroplast outer membrane (Figure 3A). Indeed, cpPD-Cy3 complexes became enveloped by the chloroplast outer membrane after they reached the chloroplast surface and were gradually pulled into the chloroplast (Figures 4B,C; Supplementary Movie 1). Although, the cpPD complex harbors a transit peptide for insertion into the chloroplast outer envelope, it has no equivalent peptide to be a substrate of the translocon at the outer and inner membranes of the chloroplast (TOC-TIC system; Jarvis and Lopez-Juez, 2013). Therefore, the cpPD complex might reach the chloroplast stroma using mechanisms distinct from the TOC-TIC system. An alternative pathway has been proposed for some chloroplast proteins. Some glycoproteins delivered by the secretory pathway are thought to be internalized to the chloroplast stroma *via* three models: fusing/budding, invagination, and pass-through (Jarvis and Lopez-Juez, 2013; Baslam et al., 2016). However, the cpPD-Cy3 complex that escapes from endocytosis is not surrounded by a lipid membrane; therefore, it appears to undergo internalization inside chloroplasts *via* an unknown mechanism. We hypothesize that the cpPD-Cy3 complex is incorporated into chloroplasts by a mechanism related to phagocytosis or pinocytosis of mammalian cells (Guidotti et al., 2017). Dissecting the mechanism underlying the uptake of cpPD-Cy3 complexes by the chloroplast would be a breakthrough in improving the integration of the peptide-DNA complex into the chloroplast.

We further examined how the cpPD-Cy3 complexes gain access to cp-nucleoids, as visualized with SYBR Green I (Figures 5, 6). The shape and location of cp-nucleoids varies as a function of growth stage and light conditions (Powikrowska et al., 2014), underscoring the importance of optimizing these growth parameters to increase the frequency of association between cpPD-Cy3 complexes and cp-nucleoids. Our growth conditions allowed the visualization of cp-nucleoids inside chloroplasts and revealed four different association stages between cpPD-Cy3 complexes and cp-nucleoids; the chloroplast without the complex (Phase I); a few complexes on the chloroplast surface (Phase II); many complexes surrounding the chloroplast surface (Phase III); the chloroplast stroma shows Cy3 fluorescence, indicative of internalization of complexes (Phase IV; Figure 6). The higher frequency of the Phase IV stage seen at 12h after infiltration (Figure 6A) would correlate with increasing accumulation of cpPD-Cy3 complexes over time. Notably, the number of cpPD-Cy3 complexes bound to cp-nucleoids is small at a given time (Figure 6B), suggesting that cpPD-Cy3 complexes may associate randomly or transiently with cp-nucleoids. Therefore, the four observed patterns likely reflect the time-dependent stages of penetration of cpPD-Cy3 complexes and the release of their DNA cargo into the chloroplast stroma.

The ultimate goal of using complexes to deliver plasmid DNA to the chloroplast genome is to allow the integration of the plasmid DNA into the organellar genome by homologous recombination, which will greatly depend on the number and localization of cp-nucleoids and the amount of plasmid DNA released from cpPD-Cy3 complexes. Because the efficiency of chloroplast transformation using the cpPD-Cy3 complex is in itself not sufficient, recombination efficiency between the chloroplast genome and the DNA cargo of the cpPD complex should be improved to capitalize on the chloroplast as a bioreactor for high-value commodities in agriculture and medicine (Verma et al., 2008; Maliga and Bock, 2011; Jin and Daniell, 2015; Adem et al., 2017). One possible improvement of recombination efficiency might be achieved with new CPPs such as BPCH7, which efficiently releases DNA after penetrating inside the cell (Chuah and Numata, 2018); the block copolymer maleimide-conjugated tetra(ethylene glycol) and poly(l-lysine; MAL-TEG-PLL; Miyamoto et al., 2019), an endosome-escaping micelle (Miyamoto et al., 2021); and new candidates from a list of 55 peptides (Numata et al., 2018) for efficient gene delivery to chloroplasts. These CPPs may be included into the current cpPD complex as conjugates (Thagun et al., 2019, 2020), which have raised the transformation efficiency of various types of plastids. Furthermore, based on the behavior of the cpPD complex in the present study, future targets to improve recombination efficiency include changing the properties of the cell wall, harnessing uptake mechanisms such as phagocytosis or pinocytosis, and increasing the colocalization of cp-nucleoids and plasmid DNA.

## Data Availability Statement

The original contributions presented in the study are included in the article/Supplementary Material, further inquiries can be directed to the corresponding authors.

## Author Contributions

KO, YK, and KN designed the study. KO, AT, MO, and KN performed the experiments. AT performed FE-SEM. All the authors analyzed the data and wrote the manuscript. All authors contributed to the article and approved the submitted version.

## Funding

This work was supported by the Japan Science and Technology Agency Exploratory Research for Advanced Technology program (JST-ERATO; grant number JPMJER1602).

## Conflict of Interest

The authors declare that the research was conducted in the absence of any commercial or financial relationships that could be construed as a potential conflict of interest.

## Publisher’s Note

All claims expressed in this article are solely those of the authors and do not necessarily represent those of their affiliated organizations, or those of the publisher, the editors and the reviewers. Any product that may be evaluated in this article, or claim that may be made by its manufacturer, is not guaranteed or endorsed by the publisher.
