# Supplementary material for: Imaging of the Entry Pathway of a Cell-Penetrating Peptide–DNA Complex From the Extracellular Space to Chloroplast Nucleoids Across Multiple Membranes in Arabidopsis Leaves
Source: Front Plant Sci. 2021 Dec 3;12:759871. doi: 10.3389/fpls.2021.759871 (PMC8678410; doi:10.3389/fpls.2021.759871)
Supplement: Supplementary file 2 [file Presentation_1.pptx]

## Slide 1
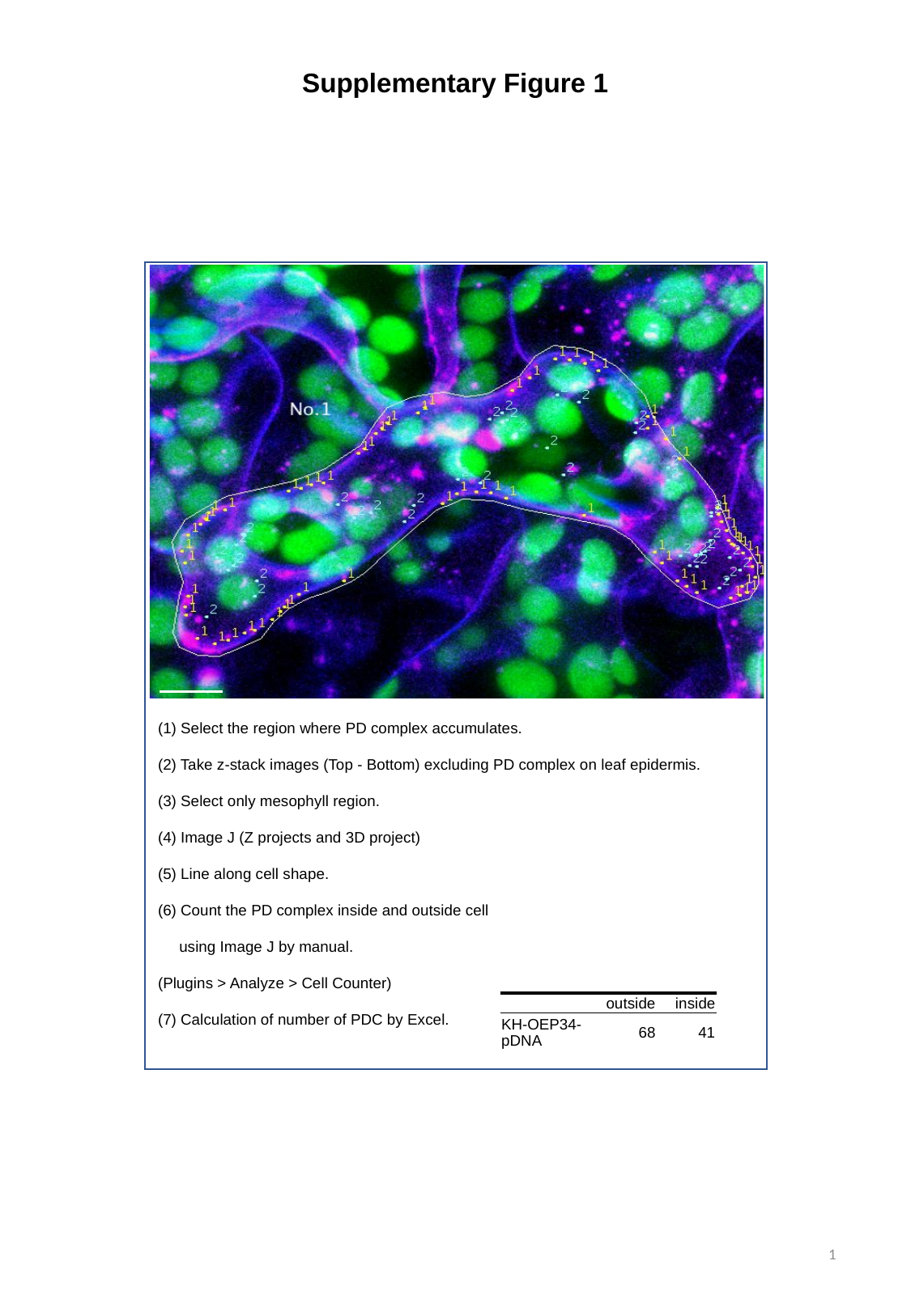

Supplementary Figure 1
(1) Select the region where PD complex accumulates.
(2) Take z-stack images (Top - Bottom) excluding PD complex on leaf epidermis.
(3) Select only mesophyll region.
(4) Image J (Z projects and 3D project)
(5) Line along cell shape.
(6) Count the PD complex inside and outside cell
 using Image J by manual.
(Plugins > Analyze > Cell Counter)
(7) Calculation of number of PDC by Excel.
| | | |
| --- | --- | --- |
| | outside | inside |
| KH-OEP34-pDNA | 68 | 41 |
1

## Slide 2
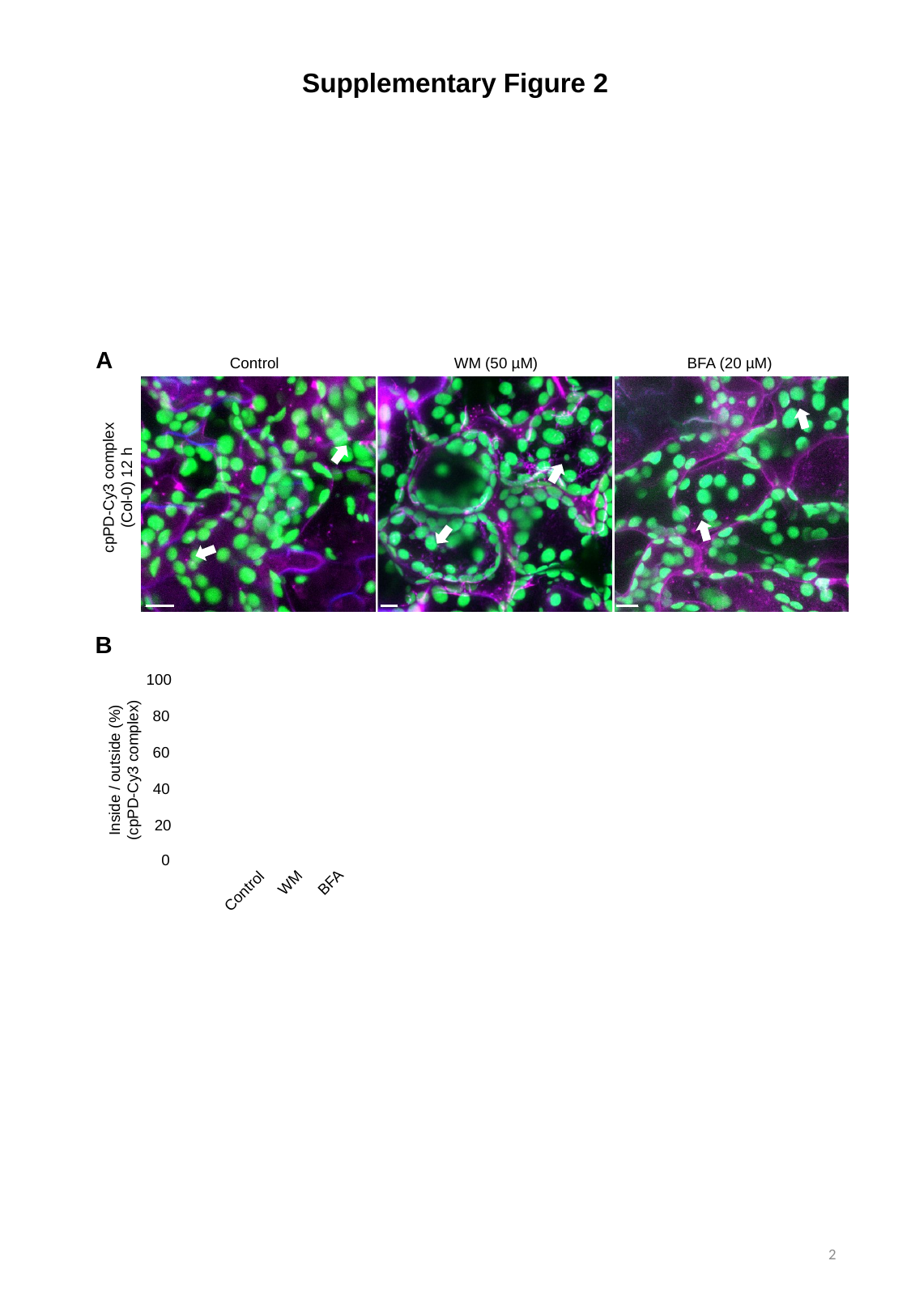

Supplementary Figure 2
A
Control
 WM (50 µM)
BFA (20 µM)
cpPD-Cy3 complex
(Col-0) 12 h
B
100
80
60
Inside / outside (%)
(cpPD-Cy3 complex)
40
20
0
BFA
WM
Control
2

## Slide 3
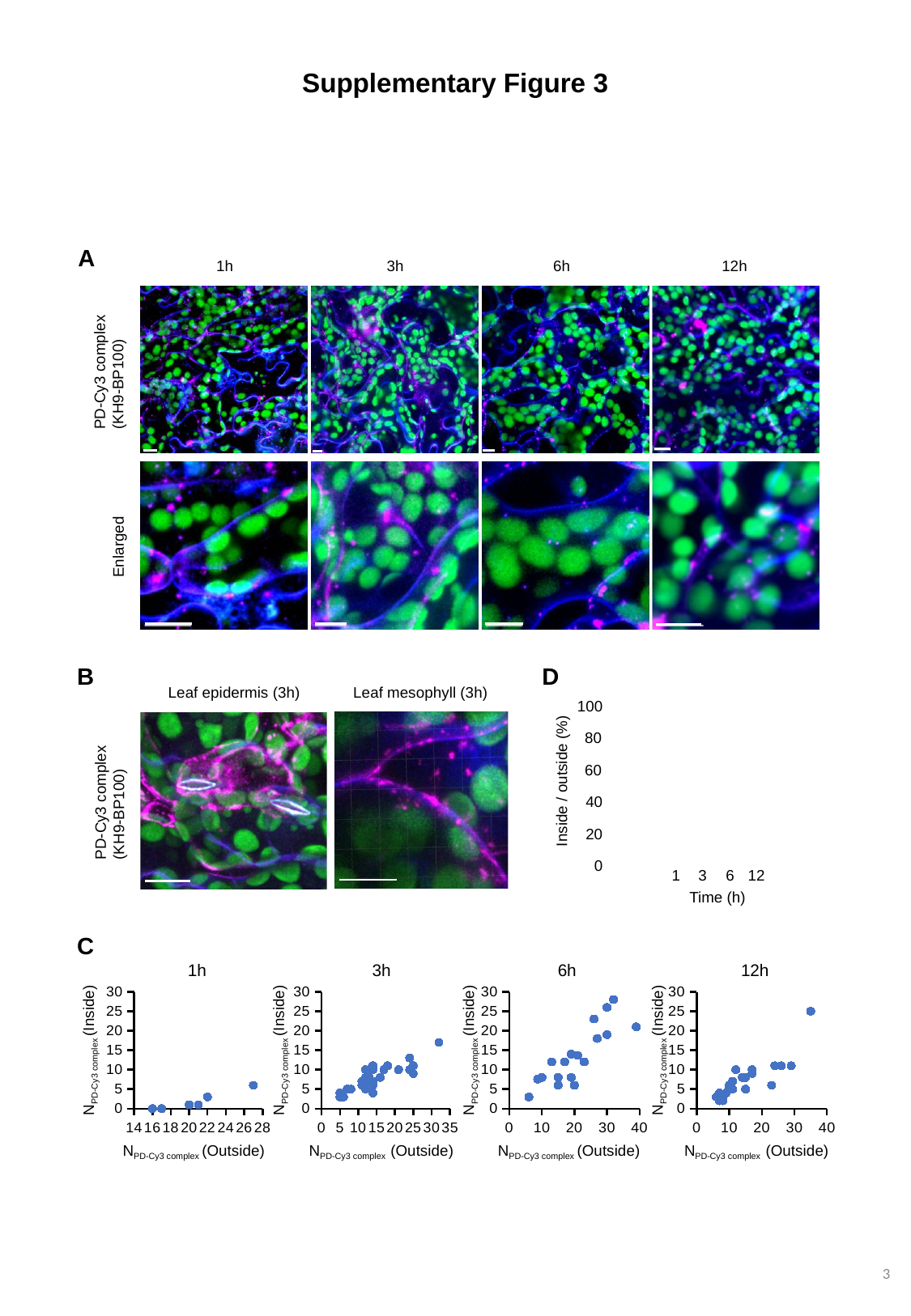

Supplementary Figure 3
A
1h
3h
6h
12h
PD-Cy3 complex
(KH9-BP100)
Enlarged
B
D
Leaf epidermis (3h)
Leaf mesophyll (3h)
100
80
60
Inside / outside (%)
PD-Cy3 complex
(KH9-BP100)
40
20
0
1
3
6
12
Time (h)
C
1h
3h
6h
12h
### Chart
| Category | in |
|---|---|
### Chart
| Category | in |
|---|---|
### Chart
| Category | in |
|---|---|
### Chart
| Category | in |
|---|---|NPD-Cy3 complex (Inside)
NPD-Cy3 complex (Inside)
NPD-Cy3 complex (Inside)
NPD-Cy3 complex (Inside)
NPD-Cy3 complex (Outside)
NPD-Cy3 complex (Outside)
NPD-Cy3 complex (Outside)
NPD-Cy3 complex (Outside)
3

## Slide 4
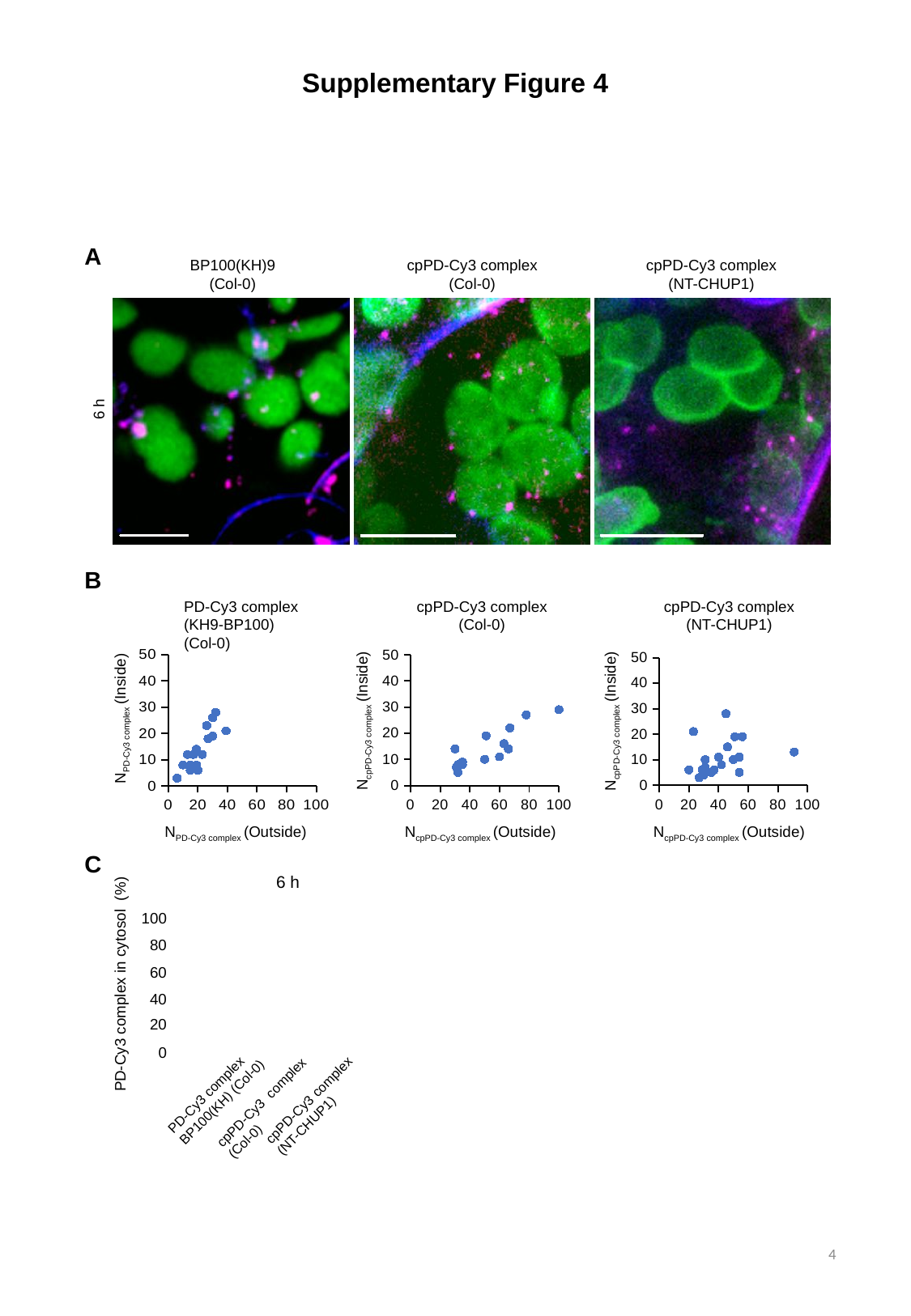

Supplementary Figure 4
A
BP100(KH)9
(Col-0)
cpPD-Cy3 complex
(Col-0)
cpPD-Cy3 complex
(NT-CHUP1)
6 h
B
PD-Cy3 complex
(KH9-BP100)
(Col-0)
cpPD-Cy3 complex
(Col-0)
cpPD-Cy3 complex
(NT-CHUP1)
### Chart
| Category | in |
|---|---|
### Chart
| Category | in |
|---|---|
### Chart
| Category | in |
|---|---|NPD-Cy3 complex (Inside)
NcpPD-Cy3 complex (Inside)
NcpPD-Cy3 complex (Inside)
NPD-Cy3 complex (Outside)
NcpPD-Cy3 complex (Outside)
NcpPD-Cy3 complex (Outside)
C
6 h
100
80
60
PD-Cy3 complex in cytosol (%)
40
20
0
PD-Cy3 complex
BP100(KH) (Col-0)
cpPD-Cy3 complex
(NT-CHUP1)
cpPD-Cy3 complex (Col-0)
4

## Slide 5
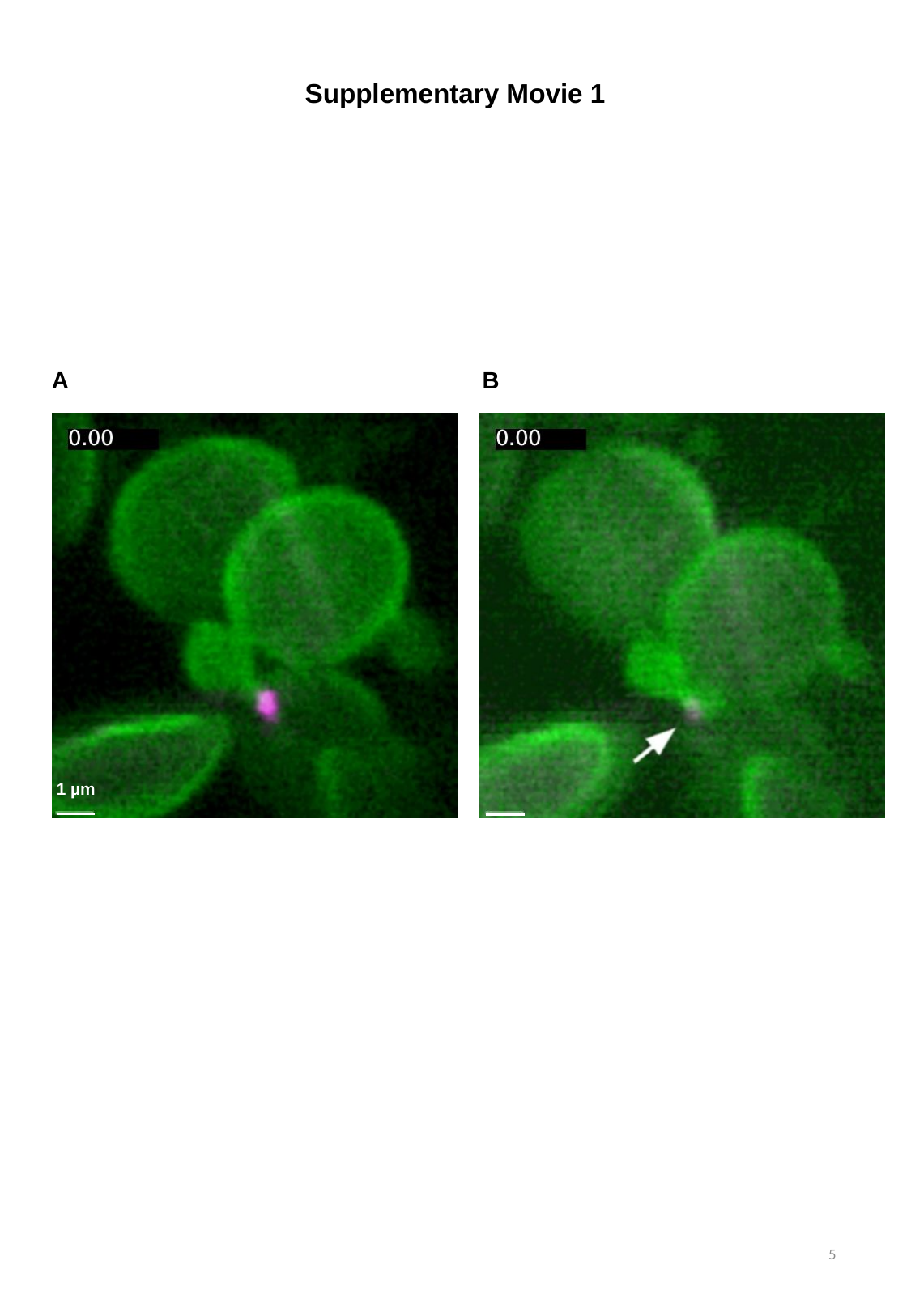

Supplementary Movie 1
A
B
1 µm
5

## Slide 6
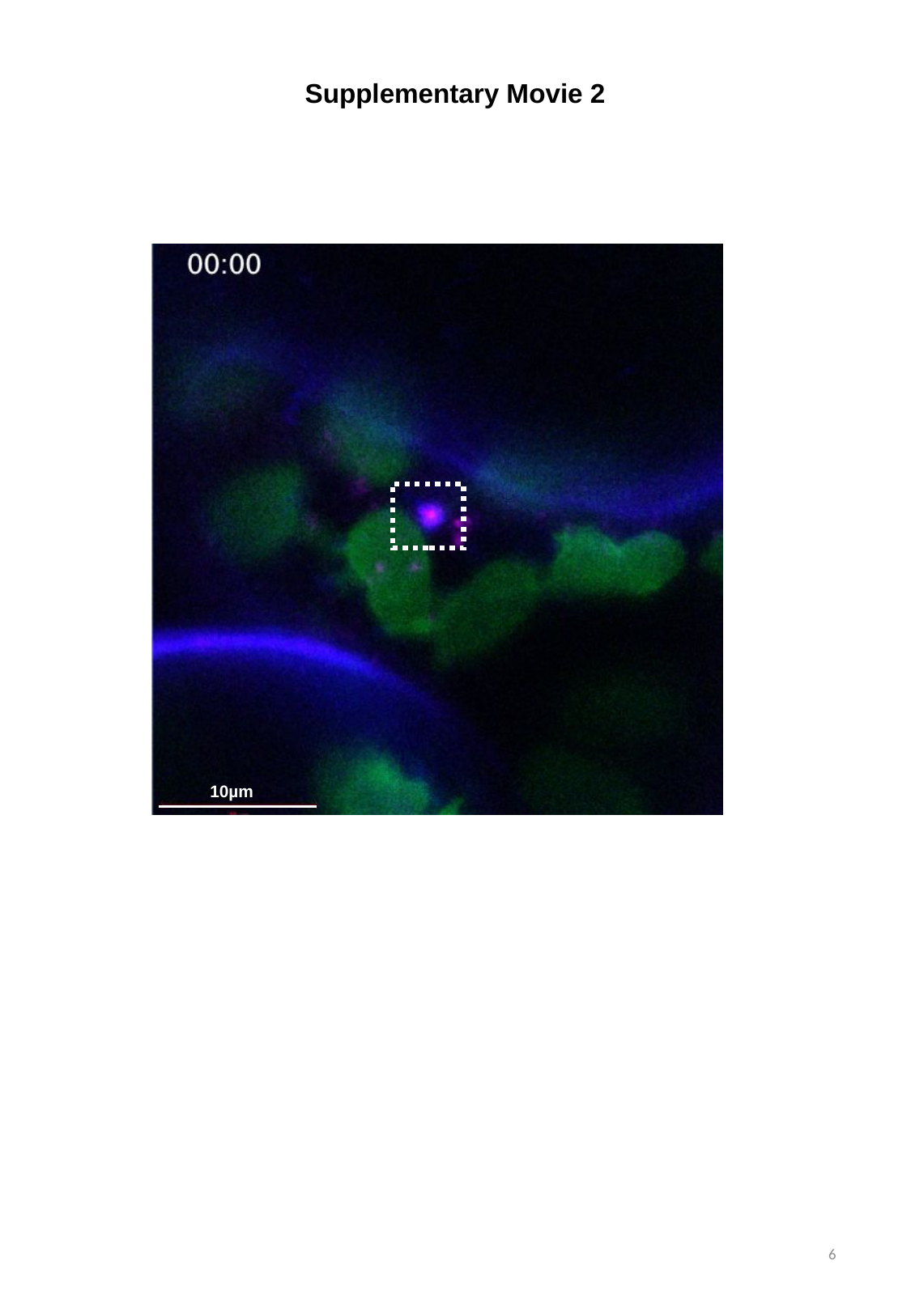

Supplementary Movie 2
10µm
6
